# Comparison between propofol and alfaxalone anesthesia for the evaluation of laryngeal function in healthy dogs utilizing computerized software

**DOI:** 10.1371/journal.pone.0270812

**Published:** 2022-07-05

**Authors:** Po-ching Pan, Christine Savidge, Pierre Amsellem, Stephanie Hamilton

**Affiliations:** Department of Companion Animal, Atlantic Veterinary College, University of Prince Edward Island, Charlottetown, Prince Edward Island, Canada; PLOS: Public Library of Science, UNITED KINGDOM

## Abstract

Laryngeal paralysis is a well-documented cause of upper respiratory tract obstruction in canines. Diagnosis of laryngeal paralysis is usually made by visual evaluation of laryngeal motion whilst patients are under a light-plane of anesthesia. However, in human studies of laryngeal function evaluation, it has been shown that subjective scoring can lead to significant interobserver variance, which may cause false diagnosis. In this study, we propose to introduce a more objective method of assessing laryngeal function using GlotAnTools and Tracker software to directly measure laryngeal motion in anaesthetized patients. Additionally, two anesthetic agents, alfaxalone and propofol, were compared in this study to assess their relative effect on laryngeal motion and thus their suitability for use in this diagnostic process. This study was a two-stage, cross-over, 1:1 randomization, with two active treatment arms. Ten beagles (10–18 months, five males and five females) were exposed to both anesthetic agents and laryngeal motion was recorded using videoendoscopy. GlotAnTools and Tracker software were applied to the recorded images to measure glottal gap area (A) and length (L). A normalized measure of laryngeal function–computed as A/L–was created, representing the "elongatedness" of the rima glottidis. The glottal gap area was significantly reduced in dogs receiving alfaxalone. This study objectively establishes that alfaxalone impacted laryngeal motion significantly more than propofol and confirms the capability of these computational methods to detect differences in laryngeal motion.

## Introduction

Laryngeal paralysis, a well-documented cause of upper respiratory tract obstruction in dogs, is characterized by impaired abduction of the arytenoid cartilages and is most commonly caused by neuropathy of the recurrent laryngeal nerve, leading to narrowing and obstruction of the rima glottidis during ventilation [1–3]. Diagnosis of laryngeal paralysis is usually made during laryngoscopy under a light plane of anesthesia [1–5]. To date, the majority of laryngeal function studies have relied on direct observation to create a subjective scoring system of laryngeal movement [6, 7] for comparison between different anesthetic agents. To the authors’ knowledge, there are no established criteria distinguishing between diseased and healthy animals. A more objective manner of measuring laryngeal motions has been investigated. These studies introduced the concept of tracking the glottal gap area [7–9]. The glottal gap area is the area created by the arytenoid cartilages and the vocal cords. During inspiration, the glottal gap area increases; while during exhalation, the glottal gap area decreases. In patients with reduced laryngeal function, the glottal gap area remains small throughout the breath cycle since the arytenoid cartilages remain immbile. Studies incorporating these measurements have also introduced a computer program for analyzing images, captured by videolaryngoscopy, to objectively measure maximum and minimum glottal gap areas [7–9]. However, these measurements are still somewhat subjective as the analysis relied on the observers to select the images showing minimal and maximal openings of the glottis [10]. Intra-observer bias has been an issue in these studies as has the ability of researchers to distinguish between small changes in glottal gap area. One study demonstrated that observers were unable to detect increases of less than 20% in glottal gap areas by direct visualization [9].

Many researchers in speech science and phonetics have examined the human glottal area via direct visual observation of the glottis using an endoscope through the nasal or oral cavity [11]. GlotAnTools is a software toolkit that has been utilized for measuring the human glottal area through high-speed video recording of the vocal folds [12]. Tracker is a computerized tool used to track the length of the glottal gap. In this study, we propose that GlotAnTools and Tracker, in combination, can be used to examine objective changes in the glottal gap area. In addition, we expect that they will be useful for developing a new more objective scoring system for laryngeal disease in dogs.

In the absence of sufficient subjects with laryngeal paralysis, we have opted to use this computerized method to compare two types of anesthetic agents commonly used in laryngeal assessments with a group of healthy animals. Animals must be anesthetized in order to evaluate laryngeal motions. Anesthesia must provide sufficient relaxation of the jaw muscles to allow opening of the mouth, without inhibiting laryngeal reflexes and inspiratory efforts [13]. Deeper planes of anesthesia result in shallow breathing or apnea and cessation of active arytenoid movement [2] leading to false positive diagnosis or misdiagnosis possibly resulting in unnecessary invasive surgical treatments. Therefore, determining anesthetics agents that are able to consistently produce the desired plane of anesthesia while having minimal effects on laryngeal motion is desirable.

Previous studies have described the effects of different anesthetic agents for evaluation of laryngeal function in dogs [6–10, 13]. Thiopental, when administered intravenously (IV), is considered to provide the best conditions for assessing laryngeal function in dogs [8]; however, thiopental is not currently manufactured in North America. In its stead, propofol is used extensively in dogs to diagnose laryngeal paralysis. Another anesthetic agent that is used for laryngeal function assessment is alfaxalone. Alfaxalone is a synthetic neuroactive steroid molecule which modulates the γ-aminobutyric acid A (GABAA) receptor causing neurodepression [14] and is similar to propofol [15]. While propofol and alfaxalone have similar mechanisms of action, there are differences in their pharmacodynamics. Alfaxalone produces a rapid induction and quick recovery times with a high safety margin, much like propofol [16–18]. However, alfaxalone is less likely to cause apnea when continuously infused at a rate of 0.07mg/kg/minute. [19]. While the effects of alfaxalone on laryngeal motion in dogs has been investigated [13, 14] these studies relied on subjective visual evaluation and the results are conflicting. In this study we compare the effects of propofol and alfaxalone on laryngeal motion in normal dogs through novel computational analyses with the aim of determining the agents’ relative suitability for the diagnosis of laryngeal paralysis.

## Materials and methods

### Animals

Ten healthy intact adult Beagle dogs, five male and five female, aged approximately 10–18 months were obtained from a commercial breeder for this study. Each dog was housed at the Atlantic Veterinary College of the University of Prince Edward Island, under the guidelines set by the College’s Animal Care Committee Code of Practice (ACC-CP-10). This study was carried out in strict accordance with the recommendations in by the Canadian Council on Animal Care (CCAC Guide, 1993) and was approved by the Animal Care Committee of the University of Prince Edward Island (Ethics certificate number: 16–012). Each dog received a physical examination prior to entering the study, including auscultation, neurologic examination, and abdominal palpation. Dogs were excluded if evidence of respiratory, cardiovascular, or systemic disease was present. A complete blood count and serum biochemistry panel was performed for each dog, to confirm general health of the animals prior to entry into the study. At the conclusion of the study, all dogs were transferred to the Department of Companion Animals for teaching purposes.

### Study design and procedures

This was a two-stage, cross-over, 1:1 randomization, two active treatment arms experimental study, and all dogs were used. Dogs were randomly assigned to either treatment sequence via its chronical order of the study participants that the first five dogs received alfaxalone first while the last five dogs received the propofol first. The order of the dogs receiving the treatment in each stage were then randomly assigned via lottery. There are five dogs in each treatment arm, providing an even distribution of each anesthetic agent at each stage. Propofol (Propofol 10 mg/ml, Baxter International Inc., Deerfield, IL, USA) and alfaxalone (Alfaxan 10 mg/ml, Jurox Pty Ltd., Kansas City, MO, USA) were the anesthetic agents used.

#### Study procedures

Intravenous catheters were placed in a cephalic vein in all of the dogs after clipping of the hair and aseptic preparation of the site. Propofol was administered at a rate of 5 mg/kg/min intravenously (IV). Alfaxalone was administered at a rate of 2 mg/kg/min IV using a syringe pump. The dose and rate of administration of the induction agents are considered to be clinically equipotent. The anesthetic agents were administered until the mouth of the dog could easily be opened for examination. A board-certified anesthesiologist (S.H.) administered both anesthetic agents and determined depth of anesthesia.

Following anesthetic induction, dogs were placed in sternal recumbency with the head held and elevated to the level of normal carriage. A 2.7 mm diameter rigid endoscope (STORZ HOPKINS® Forward-Oblique Telescope 30°, diameter 2.7 mm, length 18 cm) connected to a video DVD recorder was inserted into the mouth and over the tip of the epiglottis to a point where the entire laryngeal ostium was visible. Video recording began with the introduction of the endoscope and ended when the anesthetic plane was too light to safely continue recording; this point was identified by the same author (S.H.) performing the anesthesia. After a 14-day wash-out period, the study was repeated for all dogs using the other anesthetic agent that was not previously received.

For each anesthetic event, the final 30 seconds of the observation period was analyzed, as this was the period of lightest anesthesia prior to the completion of recording, and thus was considered most closely representative of normal awake functioning of the larynx. For each recording, at least 5 breath cycles were identified. We elected to identify the breath cycle with the greatest movement. This was based on one of the author’s (P.A.: a board-certified surgeon) opinion, that observation of one obvious movement of the arytenoid cartilages is sufficient to rule out laryngeal paralysis. For each identified breath cycle, the maximum and minimum expansion of the glottal gap were identified using GlotAnTools (GlotAnTools, Department for Phoniatrics and Pedaudiology of the University Hospital, Erlangen, Germany) and Tracker (Tracker version 4.96; Douglas Brown, Open Source Physics) software. At each maximum and at each minimum, the glottal gap area (A) and length of the glottal gap long axis (L) were measured. Both of the software programs use the color difference of the glottal gap area and the surrounding structures such as arytenoid cartilages and vocal folds to determine the A and L. The margin of the glottal gap area is usually dark where the surrounding tissue is bright pink. The accuracy of the software measuring the glottal gap area was then confirmed manually.

Subsequently, a normalized measure was computed as the ratio between these two measurements, represented as A/L, where smaller values indicate a greater elongation of the glottal gap along the major axis. Then, for each breath cycle, the difference between the maximum A/L and minimum A/L was evaluated. The breath cycle that had the greatest difference between maximum and minimum A/L (i.e. the greatest laryngeal movement) was used to compare between anesthetic agents.

Additionally, an author (P.A.), who was blinded to the induction agent used in each anesthetic episode, assessed the laryngeal function of each dog by direct visualization or via real-time laryngoscopy.

### Statistical analysis

The sample size of 10 study objects were determined based on budget. Since, to the authors’ knowledge, there are no prior studies investigating the same objectives, our study serves as a pilot study with exploratory purposes. Thus, no proceeding statistical power analysis was performed, nor a confirmatory conclusion drawn via hypothesis testing. Before cross-over to the alternative treatment arm, each beagle underwent 14-day wash-out period to sufficiently clear any carry-over effect. The balance of randomization between anesthetic agent group across two study stages will be assessed by the median and range of the age and weight of the 10 beagles, summary statistics of maximal A/L, minimal A/L, difference of the two metrics (defined as maximal A/L minus minimal A/L) overall and dividing by anesthesia agent group and the study stages.

The study result of A/L in pixel length for each beagle will also be summarized by descriptive statistics, including number of valid frames, mean, standard deviation (SD), 95% confidence interval (CI), range, maximum (max) and minimum (min) of the A/L value (in pixel length) at each study stage with corresponding anesthesia agent given. More specifically, A/L in pixel length for each beagle will be plotted for each dog by treatment.

## Results

All dogs completed the study procedures without complications. The median age was 11.5 months (range 10–18 months) and the median weight was 9.6 kg (range 8.7–11.3 kg). General physical and respiratory examinations were unremarkable for all dogs prior to anesthesia. No significant abnormalities were detected with the pre-anesthetic bloodwork (complete blood count and serum biochemistry panel) results.

**Fig 1:**
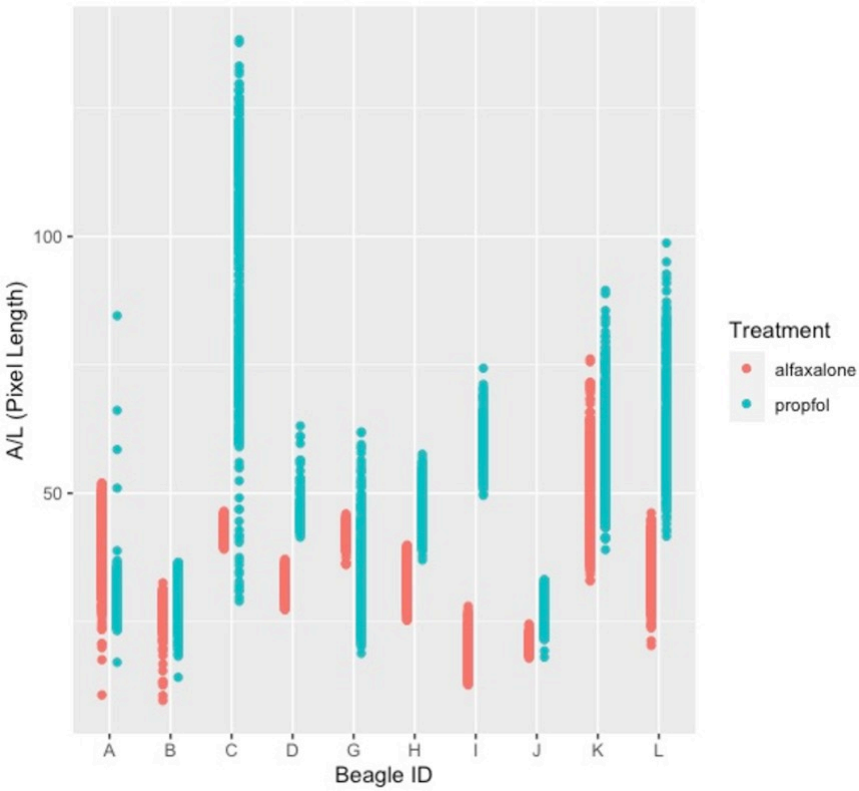
Bar chart for the variations (A/L) between dogs and different agents. The red bars indicate dogs receiving alfaxalone, and the blue bars indicate dogs receiving propofol.

An average of more than five breath cycles in the final 30 seconds were recorded during the observation period. Laryngeal paralysis was not diagnosed in any animal. Paradoxical arytenoid movements were also not observed during the videotaping periods. Table 1 summarized the A/L values in pixel length for each dog by treatment and study stage. Table 1 summarizes the beagles’ overall characteristics, maximum of A/L (A/L Max), minimum of A/L (A/L Min), difference of maximum of A/L and minimum of A/L (A/L Max–A/L Min). As presented in the Table 1. The difference of maximum of A/L and minimum of A/L (A/L Max–A/L Min) of the treatment group with propofol is bigger than the treatment group with alfaxalone. Table 2 summarizes the study design by stage, anesthetic agent group, distribution of each gender by stage and anesthetic agent group, descriptive statistics, including mean and standard deviation within each anesthetic agent group at respective stage, of maximum of A/L (A/L Max), minimum of A/L (A/L Min), difference of maximum of A/L and minimum of A/L (A/L Max–A/L Min). The table shows the equal distribution of each gender by stage and anesthetic agent group. The difference of maximum of A/L and minimum of A/L (A/L Max–A/L Min) of the treatment group with propofol remain bigger than the treatment group with alfaxalone in both stages. Fig 1 showed the variations (A/L) between dogs and different agents.

**Table 1:** Summary of study objects and study outcome of A/L values.

Characteristics		
Gender	5 / 5	
Female / Male		
Age in Months	11.5 (10–18)	
Median (Range)		
Weight in kgs	9.6 (8.7–11.3)	
Median (Range)		
Anesthetic agent	Propofol	Alfaxalone
A/L Max	70 (32)	40 (15)
Mean (SD)		
A/L Min	39 (31)	28 (11)
Mean (SD) (pixel length)		
A/L Max–A/L Min	31 (20)	13 (10)
Mean (SD) (pixel length)		

**Table 2:** Summary of study design and study outcome of A/L values by study stage and anesthesia agent group.

Stage	Stage 1		Stage 2	
Treatment	Propofol	Alfaxalone	Propofol	Alfaxalone
Gender–Female/Male	3 / 2	2 / 3	2 / 3	3 / 2
A/L Max	84 (32)	36 (9)	55 (29)	44 (19)
Mean (SD)				
A/L Min	48 (20)	21 (7)	30 (11)	34 (10)
Mean (SD) (pixel length)				
A/L Max–A/L Min	37 (17)	16 (8)	26 (24)	9 (10)
Mean (SD) (pixel length)				

Two statistic models were used to assess the difference in anesthetic agent effect of propofol and alfaxalone. The null hypothesis is that no difference between maximum A/L and minimum A/L during the last 30 seconds while the dogs are in a light plane of receiving either propofol or alfaxalone. For the reader’s interest, the detail of statistical analysis is described in S1 Appendix. The result suggests that propofol is more preferable than alfaxalone as it gives greater movement. However, we would also like to remind the reader that the normality assumption for the analysis model used in this appendix is hard to verify given small sample size. Thus, the conclusion should be used with caution in larger study.

## Discussion and conclusions

We conclude that propofol is superior to alfaxalone for the evaluation of laryngeal function in normal dogs because of a greater degree of laryngeal motion. Additionally, the software programs, Tracker and GlotAnTools, can be used to objectively measure the glottal area and to develop an objective scoring system, using A/L values, for evaluating laryngeal function.

The most common method of diagnosis of laryngeal dysfunction in dogs is made by visual recognition of the impaired abduction of the arytenoid cartilages by a trained clinician. Several recent studies have developed different systems for assessing laryngeal function [9, 10]. Some studies have attempted to develop an objective numerical scoring system to aid in diagnosis. However, others have argued that assessing the laryngeal function by direct visualization or via real-time laryngoscopy should still be considered the criterion standard [10].

In human medicine, vocal fold paresis, a partial motor denervation of the vocal folds causing variable degrees of compromised glottal function and dysphonia, is a comparable condition to canine laryngeal paralysis [20]. A recent study of vocal fold paresis demonstrated significant interobserver variance when subjectively evaluating laryngeal motion [21]. The study included fellowship-trained laryngologists that reviewed videostroboscopic examinations. The clinicians disagreed on what exactly they saw or interpreted. The overall low interobserver agreement regarding the presence or absence of paresis in these examinations reveals a significant limitation to this subjective method of analysis. Even when clinicians agree on the diagnosis, significant disagreement remains regarding which findings are important, despite individually high levels of confidence in the diagnosis [21].

To date, there are no large studies that identify interobserver error in canine laryngeal paralysis. Despite the fact that most studies include one or two board-certified surgeons as observers/raters [10], more studies examining interobserver error and if it is of substantial concern when diagnosing laryngeal paralysis are warranted.

Furthermore, the authors noticed that although there is widespread reliance on laryngoscopic examination in documenting laryngeal paralysis in dogs, there is currently no heuristic algorithm that exists for diagnosing laryngeal paralysis. Currently, clinical diagnosis of this disease is largely a case of “I know it when I see it” between the veterinary surgeons. This suggests a need for a standardized descriptive scheme for laryngoscopic findings in the diagnosis of laryngeal paralysis.

The subjective scoring system used in most studies was developed from the laryngeal examination described by White et al [7]. This system uses scores from 0–3 to describe no arytenoid movement at all to normal symmetric abduction and adduction of both arytenoids. However, it does not provide more specific details. Without a reliable scoring system, or, at minimum, a numerical diagnostic threshold, there will continue to be a lack of consensus regarding clinical findings. This lack of consensus prevents reliable conclusions regarding diagnosis, prognosis, treatment, and outcomes. Here, determined that a computer-driven analysis could be applied to canine videolaryngoscopy. In future studies, these computer programs could be used to establish an objective scoring system to reduce the chance for human error. In this study, the glottal areas were visualized during respiration using GlotAnTools, which is a software that has been used to produce a glottal-topogram for analyzing high-speed images of vocal folds in humans [12]. We also employed Tracker, another computerized tool that provides tracking of the length of the glottal gap. We chose these two software programs as they can be acquired free of charge and are relatively easy to use. While we determined that these programs could be applied to dogs, there are some potential limitations to the accurate application of these techniques. The videotaping angle of the glottal gap area is important as slight differences in recording angle between subjects n may lead to changes in the area being measured. Variations in epiglottal structure between individuals could also lead to variability in measurements. We limited the impact of these variables by analyzing a the glottal area divided by the length. By using this value, the mathematical impact of the videotaping angle becomes insignificant to the results. Furthermore, the viewing angle issue also exists when a surgeon evaluates the laryngeal area directly using a laryngoscope. To minimize this issue and to further standardize the evaluation process, a device to secure the patient’s head position in relation to the videolaryngoscope may be helpful. Further study to investigate this option could be considered.

To the best of our knowledge, no previous studies in veterinary medicine have utilized computerized software to determine the variation of the glottal gap area. Using these computerized tools, this study provided numerical, objective measurements for the evaluation of laryngeal motion. These measurements then allowed for the objective comparison of the effect of two injectable induction agents on laryngeal function. Further use and investigation of these software packages could lead to the development of a numerically-driven measurement system. This type of system could provide a means of communicating data between research groups as well as providing a more accurate means of following a patient’s clinical progression over time. We recognized this methodology cannot on its own provide an indication as to whether unilateral laryngeal dysfunction exists. While this is a notable limitation, the general consensus is that patients with unilateral laryngeal dysfunction will not benefit from unilateral lateralization [22] of the laryngeal cartilage, which is the most common surgical treatment in laryngeal paralysis patients. However, the more detailed information from video laryngoscopy as described in this paper can still improve the sensitivity and specificity in diagnosing this condition. In particular, there is potential to lateralize the use of GlotAnTools to separately evaluate the left or right side of the glottal area. Moreover, the combination of GlotAnTools and Tracker can process large amounts of data for more detailed analysis from the videos. The large amounts of data could be used to create a cumulative A/L measurement system. The cumulative measurement system could be improved upon in future studies.

The current study was not able to provide a true control group since all of the assessments needed to be performed under the influence of the anesthetic agent(s). Due to the time and finance constraints, the authors did not have access to enough of diseased dogs for the study purpose. No diseased animals were included in this study. However, the methodology described here can be used in future studies assessing diseased animals. In most canine laryngeal function studies, the biggest challenge is the lack of evaluation of diseased patients. Therefore, further studies using these software programs in diseased/affected dogs are warranted. Furthermore, it may be useful to determine if the degree of the laryngeal dysfunction (the difference of maximum and minimum A/L) is correlated to a patient’s long term prognosis without surgical treatment.

Using the described measurement system, we were able to demonstrate that the metric A/L in normal dogs receiving alfaxalone shows only 50% of variability to that compared to dogs that received propofol. This result indicates that propofol has a less negative effect on laryngeal function in healthy Beagle dogs. The reduction in the glottal gap area when using alfaxalone as the anesthetic induction agent could cause a false diagnosis of laryngeal paralysis [5, 18]. Therefore, these findings support propofol as a more favorable anesthetic agent by this metric to use when evaluating normal laryngeal function in dogs. Further studies are needed to determine if this remains true in assessing dogs with laryngeal dysfunction.

It has been shown by this study that this direct measurement is sensitive enough to detect the differences between these two anesthetic agents, even among healthy dogs. In the future, a numerical diagnostic threshold for laryngeal paralysis could be created from collecting the data of a large number of diseased and healthy dogs. Further development would be required to generate a comprehensive grading system to remove the subjectiveness of observing laryngeal movement by eye, which we believe would be the ideal for limiting misdiagnosis of laryngeal paralysis.

In future comparisons, it would be ideal to include a subjective comparison of laryngeal motion using two anesthetic agents, as well as having more than one surgeon providing their evaluations. Unfortunately, there was only one surgeon available during this study period at the University of Prince Edward Island, effectively limiting the current study. Despite being a drawback of the study, the situation reflected most likely mimics staffing conditions in a veterinary clinic where diagnosis of laryngeal paralysis in a patient may occur. In most cases, there would likely only be one veterinary surgeon available at the time of the patient’s presentation. This further emphasizes the importance of creating an objective measurement system for comparing the difference in glottal gap area between the diseased and healthy animals.

In conclusion, this study objectively establishes that alfaxalone impacted laryngeal motion significantly more than propofol and confirms the capability of these computational methods to detect differences in laryngeal motion.

## Supporting information

S1 Table Original data of the variations (A/L) between dogs and different agents. (XLSX)

S2 Table Summary study result for each dog by anesthesia agent group and study stage. (DOCX)

S1 Appendix Additional statistical analysis. (DOCX)
