# Supplementary material for: Comparison between propofol and alfaxalone anesthesia for the evaluation of laryngeal function in healthy dogs utilizing computerized software
Source: PLoS One. 2022 Jul 5;17(7):e0270812. doi: 10.1371/journal.pone.0270812 (PMC9255722; doi:10.1371/journal.pone.0270812)
Supplement: S2 Table — (DOCX) [file pone.0270812.s002.docx]

| Dog | Treatment | Study Stage | Number of Valid Frames | A/L Value  (in pixel Length)  Mean (SD) | 95% Confidence Interval (lower bound, upper bound) | Max/Min (Range) |
| --- | --- | --- | --- | --- | --- | --- |
| A | alfaxalone | 1 | 845 | 40 (6) | (28, 52) | 52 / 11 (41) |
|  | propfol | 2 | 530 | 29 (5) | (19,38) | 85 / 17 (68) |
| B | alfaxalone | 1 | 544 | 27 (2) | (22, 31) | 10 / 32 (23) |
|  | propfol | 2 | 846 | 32 (3) | (25, 38) | 37 / 14 (22) |
| C | alfaxalone | 2 | 1059 | 43 (2) | (40, 45) | 47 / 39 (7) |
|  | propfol | 1 | 900 | 93 (22) | (49, 137) | 138 / 29.0 (109) |
| D | alfaxalone | 2 | 930 | 32 (2) | (27, 36) | 37 / 27 (10) |
|  | propfol | 1 | 900 | 46 (2) | (42, 49) | 63 / 41 (22) |
| E | alfaxalone | 2 | 930 | 42 (2) | (39, 45) | 46 / 36 (9) |
|  | propfol | 1 | 994 | 30 (7) | (17, 43) | 62 / 19 (43) |
| F | alfaxalone | 1 | 900 | 33 (3) | (27, 40) | 40 / 25 (15) |
|  | propfol | 2 | 930 | 48 (4) | (41, 56) | 58/ 37 (21) |
| G | alfaxalone | 2 | 930 | 20 (3) | (15, 26) | 28 / 13(15) |
|  | propfol | 1 | 735 | 61 (3) | (54, 67) | 74 / 50 (25) |
| H | alfaxalone | 1 | 745 | 21 (1) | (18, 23) | 25 / 18 (7) |
|  | propfol | 2 | 945 | 28 (2) | (24, 32) | 33 / 18 (15) |
| I | alfaxalone | 2 | 798 | 48 (8) | (33, 64) | 76 / 33 (43) |
|  | propfol | 1 | 930 | 56 (8) | (40, 71) | 90 / 40 (50) |
| J | alfaxalone | 1 | 901 | 34 (4) | (26, 41) | 46 / 20 (26) |
|  | propfol | 2 | 820 | 60 (9) | (42, 78) | 99 / 42 (57) |
