# Supplementary material for: Comparison between propofol and alfaxalone anesthesia for the evaluation of laryngeal function in healthy dogs utilizing computerized software
Source: PLoS One. 2022 Jul 5;17(7):e0270812. doi: 10.1371/journal.pone.0270812 (PMC9255722; doi:10.1371/journal.pone.0270812)
Supplement: S1 Appendix — (DOCX) [file pone.0270812.s003.docx]

**Appendix I. Additional Statistical Analysis**

For the readers’ interest, a linear mixed model to assess the difference in anesthetic agent effect of propofol and alfaxalone. More specifically, the anesthetic agent group is the only fixed effect as an independent variable, and a random intercept is included to accommodate correlation between two measurements of each dog. The dependent outcome is the difference between maximum A/L and minimum A/L during the last 30 seconds while the dogs are in a light plane of anesthesia. The difference between maximum A/L and minimum A/L is assumed to follow normal (Gaussian) distribution and thus the random error is assumed to follow normal distribution with mean zero and a fixed number of standard deviations. The random intercept is assumed to follow the normal distribution that the random error and random intercept are independent from each other (i.e. correlation equals to zero). The restricted maximum likelihood (REML) method will be adapted to estimate parameters of interest including regression coefficient of anesthetic agent effect. The null hypothesis of zero of regression coefficient of the anesthetic agent effect will be tested under two-sided significant level of 0.05 to evaluate the possible difference of anesthetic effect using propofol or alfaxalone. In this appendix, we call this model as Model 1.

To support the statistical analysis model to assume no stage effect, the same linear mixed model including the same fixed and random effect described above, with an additional stage effect as fixed effect is performed. Likelihood ratio test with degree of freedom of 1 will be conducted using chi-square test to test the null hypothesis of no difference between two statistical models. In this appendix, we call this model as Model 2.

All analyses were performed using free software R version 4.0.2 ((2020-06-22) -- "Taking Off Again" Copyright (C) 2020 The R Foundation for Statistical Computing Platform: x86_64-w64-mingw32/x64 (64-bit)).

The Model 1 including anesthetic agent effect as fixed effect and individual effect as random intercept with difference between maximum A/L and minimum A/L gives the estimation of regression coefficient anesthetic agent effect as 18.74 with standard deviation of 6.56. The estimation of the anesthetic agent effect from the REML method yields a p-value of 0.004. The null hypothesis of no anesthetic agent effect is then rejected under 2-sided significant level of 0.05. The result shows that the same dog will have 18.74 pixel length more glottal area change in mean when switching from alfaxalone to propofol that the lower and upper bounds of 95% confidence interval of the change are 13.14 pixel length and 24.34 pixel length, respectively. Additionally, REML method gives the estimation of standard deviation of random intercept and random error as 5.68 and 14.68, respectively.

Model 2 including additional stage effect as fixed effect gives deviance (defined as -2*log of likelihood value) of 162.7 while the primary analysis model to draw conclusion about anesthetic agent effect gives deviance of 164.72. Under the null hypothesis of no model difference, the likelihood ratio test with chi-square distribution of degree of freedom 1, the p-value is 0.155.

The result suggests that propofol is more preferable than alfaxalone as it gives greater movement. However, we would also like to remind the reader that the normality assumption for the analysis model used in this appendix is hard to verify given small sample size. And thus the conclusion should be used with caution in larger study.
